# Mechanistic Investigation of Electrostatic Field‐Enhanced Water Evaporation

**DOI:** 10.1002/advs.202100875

**Published:** 2021-07-26

**Authors:** Jipeng Fei, Bin Ding, See Wee Koh, Junyu Ge, Xingli Wang, Liquan Lee, Zixu Sun, Mengqi Yao, Yonghao Chen, Huajian Gao, Hong Li

**Affiliations:** ^1^ School of Mechanical and Aerospace Engineering Nanyang Technological University Singapore 639798 Singapore; ^2^ Institute of Solid Mechanics Beihang University Beijing 100191 P. R. China; ^3^ Institute of High Performance Computing A*STAR Singapore 138632 Singapore; ^4^ School of Electric and Electronic Engineering Nanyang Technological University Singapore 639798 Singapore; ^5^ School of Chemical and Biomedical Engineering Nanyang Technological University Singapore 637457 Singapore; ^6^ CINTRA CNRS/NTU/THALES UMI 3288 Research Techno Plaza Singapore 637553 Singapore

**Keywords:** Electrostatic field enhancement, in situ Raman, molecular dynamic simulation, photothermal conversion, solar steam generation

## Abstract

Investigations on external electrostatic field (EEF)‐enhanced liquid water evaporation have been reported decades ago, which suggest that molecular alignment and polarization tuned by EEF accelerating the phase change process could be responsible for EEF‐enhanced water evaporation. However, a detailed study revealing the role of EEF in altering the intermolecular and intramolecular water structure is lacking. Herein, an EEF is proved to tune water state by accelerating the thermal movement of water molecules, lowering the molecular escaping energy, and loosening the hydrogen bond structure. The detailed mechanisms and field interactions (heat and electrostatic) are investigated by in situ Raman characterizations and molecular dynamic simulations, which reveal that an EEF can effectively reduce the free energy barrier of water evaporation and then increase the evaporated water molecule flux. As a proof of concept, an EEF is integrated into an interfacial two‐dimentional solar steam generator, enhancing the efficiency by up to 15.6%. Similar to a catalyst lowing activation energy and enhancing kinetics of a chemical reaction, the EEF enhances water state tuning, lowers evaporation enthalpy, and then boosts steam generation rate with negligible additional energy consumption, which can serve as a generic method for water evaporation enhancement in water harvesting, purification, and beyond.

## Graphical abstract

External electrostatic field is found to be effective in tuning water structure by altering the intermolecular and intramolecular interactions, as suggested by in situ Raman spectroscopy. As a result, the water evaporation enthalpy is greatly decreased, and thus significantly enhances water evaporation, which could serve as a generic strategy to increase efficiency in solar steam generation and beyond.

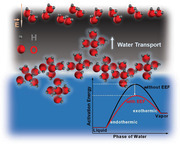

## 1 Introduction

Static electricity, normally generated by triboelectricity, electromagnetic induction, pyroelectricity, or piezoelectricity, exists ubiquitously in our daily life.^[^ ^1^ ^]^ An electric potential in an open circuit leads to accumulated charges and then results in a directional electrostatic field between electrodes. In contrast to an electric field, an electrostatic field consumes negligible energy due to the absence of electric current (directional electric charge flow). Asakawa first found that an external electrostatic field (EEF) enhanced liquid water evaporation and attributed the enhancement effect to significantly increased heat transfer of water.^[^ ^2^ ^]^ Further investigations demonstrated diverse functions of EEF on enhancement of water evaporation rate, protection of organism in water, as well as water disinfection.^[^ ^3^, ^4^ ^]^ Despite these observed interesting effects of EEF on water, the working mechanism remains elusive.

It is well known that a water stream from a tap can be pulled towards a glass (rubber) that has been rubbed with silk (fur), which is attributed to the static charge‐induced EEF that polarizes the water molecules and attracts them through Coulomb force.^[^ ^5^ ^]^ The asymmetric structure of water molecule results in strong polarizability, which would allow it to be electrically polarized and then aligned by an EEF.^[^ ^6^ ^]^ Thus, one may hypothesize that such water molecular structure alignment could alter the energy demand of water state tuning from liquid to vapor, which in turn amend the free energy at the water‐air interface. Thus, an EEF may change the intramolecular and intermolecular interactions of water molecules, and then tune water states to facilitate hydrogen bond breaking in the system. Nevertheless, direct evidence at intermolecular and intramolecular scales to support such a hypothesis is lacking.

Water evaporation has wide applications in addressing energy and environmental challenges. For instance, evaporative cooling also named evaporative air conditioner, decreases ambient temperature through water evaporation in a water vapor‐compression or absorption refrigeration cycles.^[^ ^7^ ^]^ Besides, water purification as well as electricity generation are also attracting intensive interests for environmental‐friendly energy harvesting.^[^ ^8^ ^]^ Recently, a novel interfacial steam generator has been developed for fast and efficient environmental water harvesting.^[^ ^9^ ^]^ Under natural sunlight in most areas (0.5–1.0 Sun), solar steam generator can achieve an efficiency of 85% by a heat localization structure.^[^ ^10^, ^11^ ^]^ In principle, the energy efficiency can reach 100% if 100% photo‐to‐thermal conversion efficiency and 0% heat loss are achieved (see details in Supporting Notes). Material and structural designs have been employed to promote the evaporation rate and thus to increase the total energy conversion efficiency. Alternatively, one could reduce the water evaporation enthalpy by tuning water state/molecular structure. For instance, hydrogel‐based evaporation system has achieved unprecedentedly high evaporation rate through tuning the water state.^[^ ^12^ ^]^ Notably, the overall energy efficiency of hydrogel‐based evaporator is even lower than that of two‐dimensional (2D) solar steam generator due to high heat loss arising from large water flux during evaporation. The tuned water states decrease evaporation enthalpy, in turn greatly enhance the evaporation rate. Thus, tuning water structure/state holds great promise for achieving high‐rate water harvesting.^[^ ^12^, ^13^ ^]^

Herein, we employ in situ Raman characterizations that reveal the structural change of water molecules caused by EEF‐accelerated translation and collision, expanded structural configuration (loosened intermolecular structure and increased intervals among vicinities), weakened hydrogen bond interaction, as well as smaller clusters formed by water molecules (breakage of normal tetrahedral structure). These observations verify that the EEF alters the intermolecular and intramolecular structures during phase tuning of water. Our complementary theoretical modelling results suggest that the free energy of water molecule phase transition from liquid to vapor has been significantly decreased by the EEF, which can lead to a promoted conversion efficiency. As a proof of concept, we integrate an EEF in an interfacial steam generator to significantly boost the efficiency in solar steam generation (SSG), where the EEF aligns and polarizes the water dipoles, lowers the evaporation enthalpy, and then facilitates phase change. As a result, water harvesting rate increases as the EEF strength increases until a maximum, representing a promoted SSG with 2D interfacial structure.

## 2 Results and Discussion

**Figure 1:**
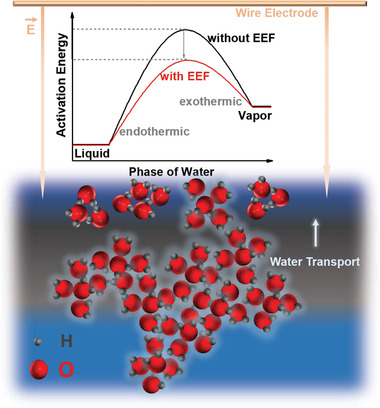
Schematic illustration of electrostatic field‐enhanced water harvesting and water state change. Water molecule structure depicts the state change under electrostatic field $\vec{E}$ exerted through the wire/mesh electrode placed just above water surface. Water exhibits unaltered structure in bulk body, but a polarized structure near the water‐air interface under the influence of electrostatic field $\vec{E}$. Inset: a plot depicting the activation (free) energy reduction induced by electrostatic field $\vec{E}$ during phase change.

Schematic setup of EEF‐enhanced water evaporation is illustrated in **Figure** **1**. At the water‐air interface, the EEF tends to polarize the water dipoles into smaller clusters through weakening the intermolecular hydrogen bond and distorting the tetrahedral structure, and further facilitates phase change of water. The phase change activation energy is decreased by the EEF with no additional energy input/consumption, similar to the catalytic effect in a chemical reaction, as depicted in the inset of Figure 1. As a result, water harvesting rate increases as the EEF strength increases.

**Figure 2:**
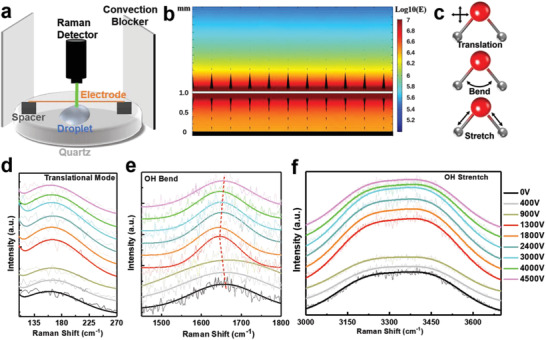
In situ Raman characterizations of interfacial water structure under an electrostatic field. a) Schematic in situ Raman setup, where the distance between wire electrode and water surface is 1 mm. b) Simulated electrostatic field distribution during in situ Raman spectrum test, field strength has a unit of V m^−1^. Color bar: electrostatic field (E) magnitude in logarithmic scale. c) Schematic illustrations of three Raman active modes. In situ Raman spectra of the d) translational mode, e) OH bend mode (dotted red line indicates peak frequency change), and f) OH stretch mode of interfacial liquid water. The laser intensity is kept constant and low to avoid heating effect. Original and fitted spectra are shown by thin and thick solid lines, respectively.

The influence of EEF on water molecules at the water‐air interface was then examined using in situ Raman spectroscopy. Bulk water exhibits complex internal structure due to the coupling of intramolecular and intermolecular movements including translation, bending, and stretching. Hydrogen bond naturally integrates two kinds of sub‐bonds which are weaker O: H nonbond (van der Waals force) and stronger intramolecular covalent bond.^[^ ^6^ ^]^ Extreme versatility of water properties is largely attributed to the hydrogen bonds, which belong to the van der Waals force and are independent of water geometric configuration.^[^ ^14^ ^]^ To detail the influence of EEF on the hydrogen bonds, we adopted the widely accepted tetrahedral structure consisting of five water molecules to investigate the behaviours of water molecules under EEF.^[^ ^5^, ^14^, ^15^ ^]^ The schematic setup of the measurement is illustrated in **Figure** **2a** (see Experimental Section and Supporting Information for details). The simulated EEF distribution indicates that an electrostatic field (>10^6^ V m^−1^) was exerted perpendicular to the water‐air interface, i.e., along the incident laser direction (green beam in Figure 2a), as depicted in Figure 2b. The influences of this EEF on the translation, bending and stretching modes (Figure 2c) of water molecule are revealed by Raman signal change upon application of EEF. In situ Raman results of three modes are shown in Figure 2d–f, and fitting of spectra are detailed in Figure S1 in the Supporting Information.

The Raman peak centered at 162 cm^−1^ represents the O—O stretching along O—H···O or hydrogen direction; and it involves the transverse and longitudinal acoustic phonons, which represent the translational mode describing the overall water molecule moving in the same direction with varying speed (depending on collisions).^[^ ^16^ ^]^ Raman peak at 162 cm^−1^ was observed under both normal and EEF‐enhanced conditions, and its position remains unchanged under varied field strengths; however, its shapes and intensities change evidently, as displayed in Figure 2d (see detailed curve fitting in Figure S2 in the Supporting Information where sub‐100 cm^−1^ spectrum was ignored as it is in the vicinity of Rayleigh line). In the absence of EEF (0 V), the 162 cm^−1^ peak has a normalized intensity and area of 53 and 6551, respectively, as summarized in Table S1 in the Supporting Information. An obvious intensified peak was observed when the external voltage was increased to 1300 V (across a 1‐mm gap), whose peak intensity (95) and area (11747) almost double, which could be ascribed to the enhanced intermolecular interaction and movements.^[^ ^17^ ^]^ Specifically, EEF‐accelerated molecular translation leads to stronger non‐bonding interaction among neighbouring water molecules promoted by Lorentz's force, resulting in an expanded structural configuration.^[^ ^3^, ^5^, ^17^ ^]^ In other words, the EEF promotes water dipoles to an activated states (increased energy) by electrical polarization, which is dependent on field strength.^[^ ^5^ ^]^ Moreover, the EEF enhancement on peak height and area starts to weaken beyond 1300 V, and finally saturates at values higher than the normal state (Table S1, Supporting Information).

Another typical vibration mode of water molecule is the OH bending, which consists of partially and fully bonded hydrogen bonds with negligible intermolecular coupling due to the small transition dipole moment.^[^ ^14^, ^18^ ^]^ The energy of a water molecule depends on its bending angle (∠H—O—H), suggesting the significance of OH bending mode in water evaporation.^[^ ^3^ ^]^ In the vapor phase, the peak centred at 1595 cm^−1^ is assigned to the OH bending mode,^[^ ^18^, ^19^ ^]^ which shows a blue‐shift of 50 cm^−1^ in the liquid state; indicating a stronger network constraining the free movement of water molecules.^[^ ^18^ ^]^ In the presence of an EEF, a red‐shift of ≈16 cm^−1^ for OH bending mode occurs from 0 to 1800 V (Figure 2e; Table S2, Supporting Information); suggesting a weakening of hydrogen bonding network and thus a reduction of restoration force on the OH bending. As such, an easier phase change from liquid to vapor is anticipated. It is worth noting that this red‐shift is opposite to the blue‐shift observed due to the increased temperature.^[^ ^19^ ^]^ Similar to the translational mode, the effect of weakening hydrogen bonding network becomes smaller (≈10 cm^−1^) from 1800 to 4500 V (Table S2, Supporting Information). Besides, a decrease of FWHM (full width at half maximum) up to 80 cm^−1^ appears from 400 to 1800 V, indicating less variety of hydrogen bonds, i.e., the ratio between partially and fully bonded hydrogen bonds is reduced.^[^ ^14^ ^]^

Compared to OH bending, OH stretching mode represents more intermolecular interactions that is highly sensitive to the ambient environment.^[^ ^19^ ^]^ Depending on the participation of a local water molecule, it can play a role as either a proton donor (D) or a proton acceptor (A) in a hydrogen bond. Thus, the local hydrogen bonding structure can be classified into DDAA, DDA, DAA and DA in liquid water under normal condition, as depicted in Figure S3 in the Supporting Information.^[^ ^14^, ^19^, ^20^ ^]^ The OH stretching mode can be deconvoluted into five Gaussian sub‐peaks from the various kinds of hydrogen bonding, but it is dominated by two sub‐peaks (93% contribution). We thus deconvoluted the OH stretching mode into two major sub‐peaks (Figure S4, Supporting Information) that were denoted as low frequency (DDAA dominant) and high frequency (DA dominant) sub‐peaks.^[^ ^20^ ^]^ As shown in Figure 2f, OH stretching peaks with various EEF possess similar shapes but distinct intensities. The fitted left and right sub‐peaks at 1800 V show up to 67% and 101% increment in height, respectively, as displayed in Table S3 and Figure S4 in the Supporting Information. Increased Raman intensity can be attributed to the increase in hydrogen bond density with EEF, due to the distortion of tetrahedral structure by strong polarization.^[^ ^3^ ^]^ Akin to the OH bending mode, too high EEF (far beyond 1800 V) decreases the effect of peak intensity enhancement, which is ascribed to the excessing hydrogen bond electrification, leading to reorientation behaviour;^[^ ^5^ ^]^ this further breaks the ordered hydrogen bond network and weakens the water structure. However, previous work demonstrated slight decrease in peak intensity and blue‐shift in frequency in OH stretching mode as temperature reached 50–60 °C, thus rising temperature can result in partial cancellation of electrostatic effect. Compared to the normal state (0 V), the ratio of sub‐peak 1 to sub‐peak 2, which reveals the percentage of various hydrogen bonds, decreases by 14% at 1800 V.^[^ ^14^, ^20^ ^]^ This ratio reduction indicates a transition towards DA‐OH dominant local hydrogen bonding structure, suggesting that external field weakens the hydrogen bonding network through changing localizing bond arrangement, which is similar to the influence of temperature changes. The favoured transition in localized connection is found to be dominant in forming smaller water cluster (n < 6) under molecular cooperative effect. Besides, the slight red‐shift of both sub‐peaks, which indicates a decrease in energy, manifests the weakening of hydrogen bond. Such a transition facilitates dissociation of molecules at the water‐air interface would result in an increase in water evaporation. To sum up, in situ Raman characterizations suggest that the influence of EEF on the interfacial water layer is to promote accelerated movement, weakened hydrogen bond, varied localizing connection, and enlarged intramolecular configuration. Thus, these observations serve as direct evidence for the strong influence of EEF on the intermolecular and intramolecular interactions of water molecules, facilitating water phase tuning.

**Figure 3:**
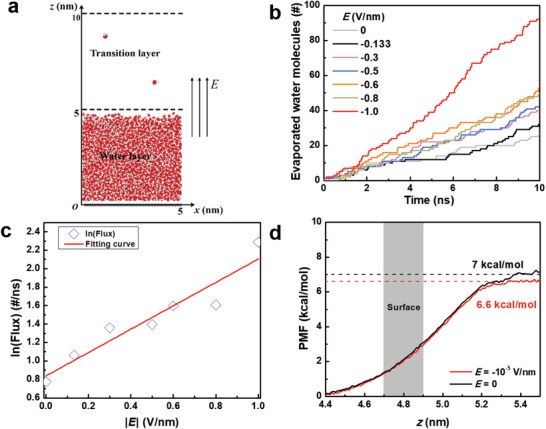
Atomistic simulations of water evaporation under electrostatic field *E*. a) Schematic of the simulation setup. A water layer with size of 5 × 5 × 5 nm^3^ is placed on top of a rigid monolayer graphene. A transition layer with thickness of 5 nm is set to distinguish the liquid state and the vapor state. A homogenous electrostatic field *E* along z direction is applied. b) Number of water molecules evaporated from the water layer under different *E* at temperature *T* = 316.45 K. c) The evaporated flux as a function of the absolute value of *E* with data points extracted from the slopes of the curves in (b). d) The PMFs of a water molecule moving across the surface under *E* = 10^−5^ V nm^−1^ and *E* = 0 at *T* = 298 K.

To rationalize the experimental findings, we conducted molecular dynamics (MD) simulations of water evaporation under different EEF (*E*). **Figure** **3a** illustrates the simulation system consisting of a water layer on a rigid graphene substrate. During simulation, water molecules that diffuse out of the transition layer are removed and counted as evaporated water molecules. Considering humidity in the experimental setting, the thickness of the transition layer (5 nm, the same as the thickness of the water layer) is larger than typical thickness (≈1 nm).^[^ ^21^, ^22^ ^]^ More details of the simulation setup can be found in Experimental Section. Figure 3b plots the number of water molecules evaporated from the water layer under different *E* (V nm^−1^) at temperature *T* = 316.45 K. The temperature and the direction of *E* are the same as in the experiment, but the magnitude of *E* is about two orders of magnitude higher to offset the limited simulation time. One can see that a higher electrostatic field can harvest more water molecules (See more details about the whole evaporation process under *E* = −0.6, 1.0 V nm^−1^ in Videos S1 and S2 in the Supporting Information). We also simulated the evaporation performance with opposite EEF, which shows that negative EEF has slightly better effect (Figure S5, Supporting Information). The slopes of the curves in Figure 3b are extracted as evaporated fluxes, and plotted in Figure 3c as a function of the absolute value of *E*. The evaporated flux increases monotonously as the absolute *E* increases. Specifically, at a given temperature *T*, the evaporated flux *J* can be rationalized by the following thermally activated Arrhenius expression

(1) $$J=A\exp\left(-\frac{U_{0}}{k_{B}T}\right)$$

where *A* is a referenced constant, *k*_B_ is the Boltzmann constant, and *U*_0_ is the activation energy for water evaporation. The value of *A* and *U*_0_ can be obtained from the simulations of water evaporation under different temperature *T* in the absence of *E* (Figure S6, Supporting Information). By a linear fit of ln(*J*) to 1/*T*, one obtains ln(*A*) = 20.63, *U*_0_ = 12.42 kcal mol^−1^. Here *U*_0_ is about 4 times larger than the activation energy for water diffusion.^[^ ^23^ ^]^ Then we imported the parameter *α* to characterize the influence from the electrostatic field *E* and replaced *U*_0_ in Equation (1) with *U*_0_ − *α*|*E*|,

(2) $$J=A\exp\left(-\frac{U_{0}-\alpha \left|E\right|}{k_{B}T}\right)$$

We used Equation (2) to fit the simulation data points in Figure 3c and obtained *α* = 0.87 kcal mol^−1^/(V nm^−1^) at temperature *T* = 316.45 K. By the same approach, we calculated *α* = 0.80 kcal mol^−1^/(V nm^−1^) at *T* = 298 K (Figure S7, Supporting Information), *α* = 0.70 kcal mol^−1^/(V nm^−1^) at *T* = 335.35 K (Figure S8, Supporting Information), and plot *α* as a function of *T* in Figure S9 in the Supporting Information. It can be observed that the EEF enhances the water evaporation at all temperatures, while a higher temperature tends to attenuate the magnitude of enhancement. As the temperature reaches the boiling point, the enhancement from the electrostatic field become negligible.

Furthermore, free energy calculations were utilized to provide a quantitative measure for the effect of the electrostatic field. The absolute value of *E* adopted here was chosen as small as 10^−5^ V nm^−1^ to mitigate the kinetic effects of water molecules and maintain equilibrium. The potential of mean force (PMF) of an arbitrary water molecule moving across the surface with and without *E* are summarized in Figure 3d. The energy barrier for water evaporation under *E* = 0 at *T* = 298 K is 7.0 kcal mol^−1^, which is consistent with previous calculations (7.4 kcal mol^−1^ at *T* = 298 K by SPC/E model,^[^ ^24^ ^]^ 6.3 kcal mol^−1^ at *T* = 298 K by experiments,^[^ ^24^ ^]^ and 6.7–7.1 kcal mol^−1^ at *T* = 300 K by TIP4P/Ew model^[^ ^22^ ^]^). Application of an EEF of 10^−5^ V nm^−1^ would deduct the energy barrier of water evaporation by 0.4 kcal mol^−1^, which plays a role of a catalyst, consistent with experimental findings.

**Figure 4:**
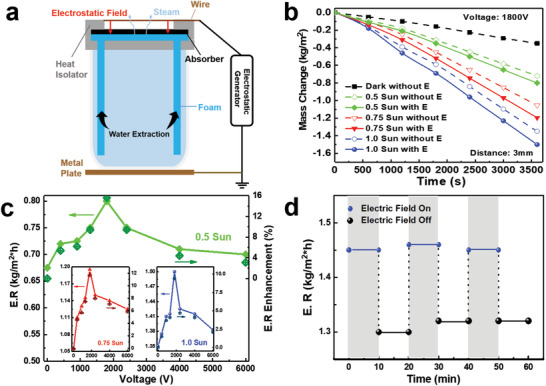
EEF‐enhanced solar steam setup and performances. a) Schematic of EEF‐enhanced steam generation setup. b) Time dependent water harvesting amount under normal and EEF enhancement conditions at different solar intensities. The distance between the metal wire electrode and water‐air interface is 3 mm. c) Evaporation performances under three different solar intensities, i.e., 0.5 Sun, 0.75 Sun (left inset), and 1.0 Sun (right inset), with different EEF strengths, where the field strength is tuned by wire electrode voltage. d) EEF enhancement sensitivity measurement by switching ON/OFF voltage supply with an interval of 10 min under continuous solar illumination.

As a proof of concept of application, we demonstrate EEF‐enhanced evaporation in SSG. A solar steam generator couples interfacial porous photothermal absorber with a heat isolation structure, localizing heat at the water‐air interface for maximized energy utilization.^[^ ^25^ ^]^ Leveraging the photothermal effect, a solar steam generator drives interfacial steam generation with solar energy.^[^ ^11^, ^26^ ^]^ Prior to enhancing solar steam generation by electrostatic field, we calibrated our previously reported solar absorber, i.e., the cellulose acetate membrane (CAM), which shows excellent photo‐thermal conversion and water transport capability (as illustrated in Figures S10–S15 in the Supporting Information). The EEF‐enhanced SSG is shown in **Figure** **4a**, where a 70‐µm‐thick copper wire electrode (see Figure S16d in the Supporting Information for a photo) is placed 3 mm above the CAM surface and a grounded metal‐plate electrode is situated at the bottom of the water tank. Water is extracted upwards through a foam pumper and then transports to the interface by capillary forces inside the CAM with porous polymeric structure. The calculated electrostatic field distribution around the copper wire electrode exhibits a directional electrostatic field, with a magnitude of 10^5^‐10^6 ^V m^−1^ depending on the voltage applied on the copper wire electrode, exerted on the water‐air interface (Figure S17, Supporting Information). An uneven electrostatic field appears from the cross‐sectional view, showing a significantly larger field between the wire electrode and the CAM absorber. Figure 4b displays the accumulated mass change due to water evaporation under different solar intensity with EEF (1800 V across a 3‐mm gap). As the solar intensity rises from 0.5 Sun to 1.0 Sun, the overall mass change dramatically increases. The larger mass change with EEF (solid lines) than that without EEF (dashed lines) indicates a higher conversion efficiency with the same solar energy input. As shown in Figure 4c, the evaporation rate (E.R.) increases as the EEF increases until a peak appearing around 1800 V, after which the EEF enhancement is weakened; consistent with Raman results in Figure 2.

The energy conversion efficiency of this SSG was calculated as $\eta =\frac{R\times h_{lv}}{I\times t}$ (see Experimental Section for details). Since EEF is found to tune the water molecular structure, the evaporation enthalpy could change; thus, a control dark experiment was conducted to evaluate the water evaporation enthalpy. As displayed in Figure S18 in the Supporting Information, the energy barrier for water escaping from surficial layer into air was lowered by EEF, leading to the enhanced evaporation performance. Consequently, the water evaporation enthalpy is estimated to decrease from 2256 to 1511 J g^−1^ by EEF (Supporting Information). Notably, the high harvesting rate promotion achieved under 0.5 Sun illumination (Figure 4c) suggests that efficient water harvesting in cloudy weather is possible with EEF enhancement. To verify the enhancement of EEF, the dynamic response of the SSG process was recorded in Figure 4d. With an interval of 10 min, a fast increase and decrease of E.R. were observed when the EEF was switched ON and OFF sequentially. The excellent reproducibility of EEF enhancement suggests that EEF‐enhanced SSG is effective and stable. It is worth noting that the two fields coupled within this solar steam generator, i.e., thermal and electrostatic fields, have opposite effect on modifying the structure of water molecules. The thermal field tends to increase the entropy of water within 270 to 340K while the electrostatic field tends to decrease the entropy of water by aligning water dipoles.^[^ ^3^, ^27^ ^]^ Experimentally, one can see that EEF enhancement is stronger at lower temperature (under 0.5 and 0.75 Sun) than that at higher temperature (under 1.0 Sun) (Figure S19, Supporting Information), which can be attributed to the competing influence of thermal and electrostatic fields; as revealed by MD simulation in Figures S7–S9 in the Supporting Information. This observation suggests the EEF enhancement can be an effective tool to enhance SSG in regions or weathers with low solar intensity.

**Figure 5:**
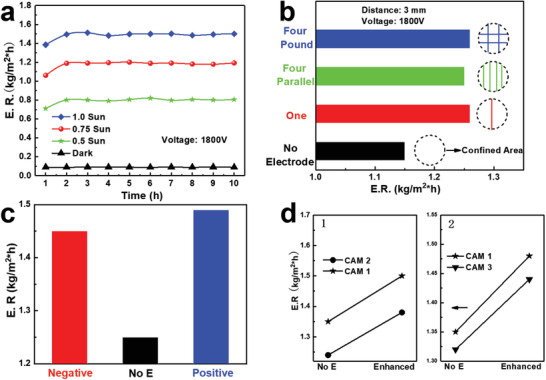
Stability test and various factors influencing EEF enhancement. a) 10‐h stability test under continuous illumination with EEF enhancement, where the duration is consistent with the measured sunlight duration in Figure S15 in the Supporting Information. b) The influence of wire electrode configuration EEF enhancement under 0.75 Sun, where the dotted circle represents confined evaporation area for precise evaluation of E.R. c) The influence of field direction on EEF enhancement. d) The influence of CAM component on EEF enhancement. Panel 1 (left) compares two samples with different Li‐MXene contents but the same thickness, and Panel 2 (right) compares two samples with the same Li‐MXene: cellulose acetate ratio but different thicknesses.

Next, we evaluate various factors that might influence the EEF enhancement. **Figure** **5a** depicts the stability of E.R. of SSG under different solar intensities, where the dark condition measurement serves as a reference on the humiture effect. The successive enhanced water evaporation rate over 10‐h continuous operation suggests excellent stability of the EEF enhancement effect. Besides, the influence of wire electrode configurations and density are compared among four designs, i.e., one straight wire electrode (red bar), four parallel wire electrodes (green bar), 2 × 2 grid wire electrode (blue bar), and absence of wire electrode (black bar), as shown in Figure 5b. Negligible difference was found with distinct wire electrode configurations and densities, and all configurations efficiently enhanced the SSG. This might be attributed to the large distance (3 mm) from the wire electrode to the water‐air interface, resulting in the negligible influence of wire electrode configuration on the electrostatic field distribution at the water‐air interface that determines the EEF enhancement effect on SSG efficiency. Moreover, the influence of the direction of EEF on SSG was also examined, and slight difference on SSG was observed when the direction of EEF was reversed, as shown in Figure 5c (with the normal SSG without EEF as the reference). EEF with negative direction appears more effective in enhancing water evaporation, which qualitatively agrees with our MD calculation (Figure S5, Supporting Information), as well as earlier report.^[^ ^3^ ^]^

To further confirm that water‐air interface plays the most important role in SSG, we compared the CAM with different compositions and thicknesses, as depicted in Figure 5d. Since Li‐MXene is metallic, more electrostatic field screening inside CAM would occur when the Li‐MXene content in CAM increases. Sample CAM2 contains 50% less Li‐MXene content than sample CAM1 (with the same thicknesses), and similar enhancements in SSG performances are observed, as shown in the left panel of Figure 5d. This comparison suggests that the electrostatic field inside CAM has a minor contribution to EEF‐enhancement in SSG. Moreover, though sample CAM3 has doubled thickness (Figure S20, Supporting Information) and thus the doubled length of water transport path compared to sample CAM1 (with the same composition), the enhancement in SSG performances is comparable, as displayed in the right panel of Figure 5d. This comparison suggests that the water transport path inside CAM plays a minor role in EEF‐enhancement in SSG. Overall, these observations indicates that the EEF enhancement in SSG is dominated by the electrostatic field at the water‐air interface, which influences the state tuning of interfacial water molecules. Moreover, operando microscopy images (Figure S16a–c, Supporting Information) show negligible change of surface topography of CAM under various EEF, suggesting that the influence of CAM structure change can be neglected. These comprehensive comparisons prove that the interfacial water polarization induced by EEF dominants the enhancement, which results in lower free energy and facilitates the water phase change. Such an EEF‐enhanced water evaporation at water‐air interface could find other applications such as evaporative conditioner.

## 3 Conclusion

In summary, the enhancement of state tuning of interfacial water molecule has been achieved by EEF. Electrostatic force polarizes water dipoles and affects translation and vibration modes simultaneously, resulting in a reduction of the energy barrier for water phase change. Water structure under strong EEF shows several characteristics as revealed by in situ Raman spectroscopy: 1) accelerated molecular movement and stronger collision possibility promoted by polarization and Lorentz Force, 2) weakened hydrogen bond strength as reflected by a red‐shift of frequency, 3) distorted tetrahedral structure with varied localizing hydrogen bonding network assisted by EEF, and 4) expanded structural configuration resulting from loosened hydrogen bond. These in situ Raman spectra reveal the mechanism of enhanced water evaporation due to water structural change under EEF. Theoretical investigation suggests that the free energy barrier of water evaporation is effectively reduced by the external electrostatic field, and thus increases the evaporated water molecule flux. Significant field interaction between heat and electrostatic is studied by the theoretical simulation, which provides further guidance for achieving better enhancement. Moreover, an EEF is integrated into a solar steam generator and leads to enhanced water evaporation performance with negligible extra energy consumption. This work reveals the mechanism of water state tuning by an external electrostatic field, and showcases an application in a solar steam generator, and thus opens a new route for improving water evaporation that could find many engineering applications beyond water harvesting.

## 4 Experimental Section

### In Situ Raman Characterization

Figure 2a shows the setup for in situ Raman test (WITec alpha300 R Raman spectrometer) employing quartz glass as the substrate to reduce the undesired background signal. Before characterization, the system was calibrated with Si peak at 520 cm^−1^. A single water droplet was placed in the middle of the quartz glass, which was protected by a convection blocker. A copper wire electrode was supported by two spacers to keep a 1‐mm distance from the water surface. During the test, laser intensity (<1 mW, 532 nm) and other parameters were kept unchanged with only the external voltage on the electrode varying. Both temperature and humidity were well controlled in the characterization room.

### Computational Method

To complement the experiments, molecular dynamics (MD) simulations were performed by using the large‐scale atomic/molecular massively parallel simulator (LAMMPS).^[^ ^28^ ^]^ The simulation system consisting of a rigid graphene substrate and a water layer was generated by the Visual Molecular Dynamics.^[^ ^29^ ^]^ The constructed water layer was 5 × 5 × 5 nm^3^ in size and contained 3730 water molecules. The rigid non‐polarizable TIP4P/Ew model was employed for water molecules, with charge distributions of +0.5242*e* for a hydrogen atom and −1.0484*e* for an oxygen atom.^[^ ^30^ ^]^ This water model has been validated for liquid‐vapor phase changes.^[^ ^22^, ^30^ ^]^ The particle‐particle particle mesh (PPPM) method was employed to compute the long range electrostatic interactions.^[^ ^31^ ^]^ The SHAKE algorithm with tolerance of 1.0 × 10^−4^ was adopted to maintain the rigidity of water molecules. The Lennard‐Jones (LJ) potential with a cut‐off of 1.2 nm was used to describe the non‐bonded van der Waals interactions in/between the graphene substrate and water molecules. All LJ parameters are listed in Table S4 in the Supporting Information. The system was first relaxed at a constant temperature of *T* = 316.45 K for 100 ps under a canonical NVT ensemble. Then an external electrostatic field was applied, and the simulation system was allowed to relax for 10 ns. Any water molecules with position coordinate *z* > 10 nm (that diffused out of the transition layer) were removed from the system. Periodic boundary conditions were imposed in the *x*‐ and *y*‐directions of the simulation system. The integration time step was taken as 1 fs.

In further support of the experimental findings, the potential of mean force (PMF) of an arbitrary water molecule moving across the water surface was also calculated by the umbrella sampling method. In this calculation, the temperature was maintained at room temperature (298 K). Two scenarios were chosen with the external electrostatic field taken as *E* = 0 and *E* = −10^−5^ V nm^−1^, respectively. To obtain the free energy profile, 20 independent windows were generated with a step size of 0.1 nm between neighbouring windows along the evaporation direction from *z* = 4.0 nm to *z* = 6.0 nm. The coordinate *z* of the water molecule was restrained relatively by a harmonic spring (1000 kJ mol^−1^ nm^−2^). Each window ran for 6 ns, with coordinates collected every 1 ps after 2 ns. Then the unbiased PMF was estimated from all biased probabilities of each window by the weighted histogram analysis method (WHAM) with a tolerance of 10^−6^.^[^ ^32^ ^]^

### MXene Fabrication

MAX phase Ti_3_AlC_2_ was purchased from Y‐Carbon Ukraine. Li‐MXenes were prepared with a typical MILD process with the etchant consisting of 100 mL of 6 M HCl (Sigma Aldrich, 37%) with 3.96 g LiF (Sigma Aldrich, 99.995%). The duration of etching was 1 week to ensure the material was etched completely. The etched materials were retrieved after cleaning via centrifugation at 5000 rpm for 10 min and replacing the supernatant with DI water for at least 5 cycles until the pH value reached above 5.0. The powder was obtained after drying in vacuum at 60°C for 15 h.

### CAM Fabrication

Proper amount of Li‐MXene was treated twice with ultrasonication and grinding to avoid clustering. Cellulose acetate and poly(ethylene glycol) (PEG) powder were mixed with a ratio of 4:1 for the polymeric structure. PEG was added for tuning the hydrophilicity. The mixed powder was dissolved into a mixed solvent with N‐Methyl‐2‐pyrrolidone (NMP) and acetone (ratio: 1:7) for better gelation during the phase transition process. After that, Li‐MXene was slowly added into the mixed solution followed by stirring for 2 h at 300 rpm. Then, the solution was left at room temperature to remove bubble for 30 min without stirring. Molding methods were employed in the experiment to precisely control the membrane sizes. Molded solution after 1‐h evaporation under room temperature was transferred into cold water for phase transition. Components of three samples (CAM1, CAM2, and CAM3) were: 1) 0.8 g cellulose acetate, 0.2 g PEG, 50 mg Li‐MXene; 2) 0.8 g CA, 0.2 g PEG, 100 mg Li‐MXene; and 3) 0.4 g CA, 0.1 g PEG, 25 mg Li‐MXene.

## Conflict of Interest

The authors declare no conflict of interest.

## Supporting information

Supporting Information

Supplemental Video 1

Supplemental Video 2

## Data Availability

The data that support the findings of this study are available from the corresponding author upon reasonable request.
