# Supplementary material for: Mechanistic Investigation of Electrostatic Field‐Enhanced Water Evaporation
Source: Adv Sci (Weinh). 2021 Jul 26;8(18):2100875. doi: 10.1002/advs.202100875 (PMC8456210; doi:10.1002/advs.202100875)
Supplement: Supplementary file 1 — Supporting Information [file ADVS-8-2100875-s003.pdf]

© 2021 Wiley-VCH GmbH

ADVANCED SCIENCE
Open Access

Supporting Information

for *Adv. Sci.,* DOI: 10.1002/advs.202100875

Mechanistic Investigation of Electrostatic Field-Enhanced Water Evaporation

Jipeng Fei, Bin Ding, See Wee Koh, Junyu Ge, Xingli Wang, Liquan Lee, Zixu Sun, Mengqi Yao, Yonghao Chen, Huajian Gao, and Hong Li**

Supporting Information

Mechanistic Investigation of Electrostatic Field-Enhanced Water Evaporation

Jipeng Fei,† Bin Ding, † See Wee Koh, Junyu Ge, Xingli Wang, Liquan Lee, Zixu Sun, Mengqi Yao, Yonghao Chen, Huajian Gao,* Hong Li*

J. Fei, S. W. Koh, J. Ge, L. Lee, Z. Sun, M. Yao, Prof. H. Li, Prof. H. J. Gao
School of Mechanical and Aerospace Engineering

Nanyang Technological University, 639798, Singapore

Email: ehongli@ntu.edu.sg

Email: huajian.gao@ntu.edu.sg

Dr. B. Ding, Prof. H. Gao

Institute of High Performance Computing

A*STAR, Singapore 138632, Singapore

Dr. B. Ding

Institute of Solid Mechanics

Beihang University

Beijing 100191, P. R. China
Dr. X. Wang, Prof. H. Li
School of Electric and Electronic Engineering

Nanyang Technological University, 639798, Singapore

Dr. Y. Chen

School of Chemical and Biomedical Engineering

Nanyang Technological University, 637457, Singapore

Prof. H. Li

CINTRA CNRS/NTU/THALES

UMI 3288, Research Techno Plaza, 637553, Singapore

† equal contribution

ORCID

Huajian Gao: 0000-0002-8656-846X

Hong Li: 0000-0002-6975-7787

Supporting Notes

Characterizations

SEM images were obtained from JEOL 7600L. Visible- NIR absorbance was tested by spectrometer coupled with an integrating sphere (Shimadzu, UV-1800). Heat distribution map was obtained by an IR camera (NEC F30W). Atomic force microscopic images were taken by Park Systems NX10. Contact angle test was conducted by LMS Scientific Solution Sdn Bhd OCA 200. Microscopic images were taken by OLYMPUS BX53M series. *In-situ* Raman signal was characterized by Raman spectrometer (WITEC alpha300 R confocal Raman system) with customized sample stage. Before testing, the system was calibrated with Si peak at 520 cm^{-1} to keep the measurement consistent.

An OHAUS- Pioneer Series balance was used to record the weight change during harvesting. With copper a wire connected as the electrode, an electrostatic generator (TREK 610-E) was employed to generate a strong external electrostatic field. Simulated sunlight was generated by a solar simulator (LCS-100) for continuous illumination, and the light direction was tuned by a reflector. The solar power meter employed was technical series from Silicon Lab. Desalination performance was measured by Hang Zhou, Qiwei Equipment-DWS 51.

Water Harvesting Performance Measurement

Water harvesting performance was conducted under controlled humiture. An electrostatic field was exerted externally by a copper wire above CAM. A high resolution digital balance was used for weight tracing under continuous illumination. Precise energy input control was calibrated by a solar power meter. An area of 5 cm* 5 cm at the center of illumination from the solar simulator was defined by an opening on a PMMA plate to maximize the light uniformity. Also, an accurate calculation of evaporation area will lead to reliable result.[1] The slope of designed water harvesting device was used for collecting the generated steam. A metal plate was placed at the

bottom of the device and connected with a ground electrode. Under continuous illumination, the weight change was traced to get the evaporation rate, and the temperature change was measured by the IR thermometer simultaneously. Energy conversion efficiency can be derived by equation: $\eta = \frac{R \times h_{lv}}{I \times t}$, where R is the evaporation rate, h_{lv} is the liquid to vapor latent heat, I is the solar power density, and t represents time period. Balance enclosure hood was closed to avoid any ambient air movement during water harvesting. A transparent container made from PMMA was designed for water collection. For energy conversion efficiency calculation, we have conducted a dark experiment to reevaluate the water evaporation enthalpy, details are shown in Supporting Information- Evaporative Water Enthalpy Evaluation.

Evaluation of Photo-thermal Absorber and Setup for Water Harvesting

The photothermal absorber used in this work was Li-Mxene interpenetrated cellulose acetate membrane (CAM) similar to that employed in our previous report.[2] The hierarchical porous structure of the polymeric membrane greatly facilitated the water phase tuning and heat localization (**Fig. S1**). During the wetting process, polymeric structure underwent swelling, resulting in a lumpy surface topography (**Fig. S2**) and thus an increased water-air interface area. Besides, CAM had a very hydrophilic surface, facilitating the water transport across the CAM membrane (**Fig. S3**). The CAM surface temperature increased to 45.7 ℃ under 1.0 Sun illumination due to the high solar absorbance (**Fig. S4e, S5**). The lower temperatures on CAM surface under 0.5 Sun (37.5 ^{o}C) and 0.75 Sun (40.6 ^{o}C) indicated a solar-power-density-dependent heat generation in CAM (**Fig. S4a, S4c**). Besides, excellent heat localization was revealed by the cross-sectional heat map (**Fig. S4b, S4d, S4f**). With controlled room temperature (23 ℃) and humidity (70%-75%), an overall energy utilizing efficiency of the CAM absorber reached 84.6%, 88.1% and 84.6% under 1.0, 0.75 and 0.5 Sun, respectively. This suggests stable performance among various solar intensities. The real-time sunlight intenisity on our lab rooftop (Singapore N 1°20’54.4”; E 103°40’59.3”, March 2020) was recorded as a reference

(**Fig. S6**), which revealed that natural solar intensity is often lower than 1.0 Sun; thus, efficient energy conversion with low solar intensity is crucial for practical application.

EEF-Enhanced Solar Steam Generation

Lastly, we tested the EEF enhancement effects in a water harvesting setup, as shown in **Fig. S21**. The water harvested from high salinity seawater meets both WHO and EPA standards of drinkable water (**Fig. S22**).[3] Besides, a complete removal of pollutant (methyl orange) was achieved in the harvested water from the polluted wastewater (**Fig. S23**). Additionally, neutral water was obtained from acidic or alkaline water, as presented in **Fig. S24**. These tests show that our SSG device has high flexibility, multifunctionality, and excellent resistance to harsh environment. Importantly, the EEF-enhancement effect in SSG is versatile in various applications. Therefore, improved design to further increase the effective EEF strength at water-air interface could further enhance the EEF effect on water steam generation.

Energy Efficiency Limitation of 2D Solar Steam Generation System

The overall process of solar steam generation can be described as applying solar power in water phase changing. From energy conversion equation, efficiency (η) depends on the input solar power density as well as the corresponding evaporation rate (R). Here, the efficiency limitation represents the 100% utilization of solar power into vapor generation with normal latent heat demand in phase change. Thus, each solar intensity has a certain limited evaporation rate under normal state (no contribution from external factors). However, energy loss during both photo-to-thermal and systematic heat transfer are inevitable, which leads to efficiency drop from 100%.[2, 4] Based on the equation $\eta = \frac{R \times h_{lv}}{I \times t}$, h_{lv} is a key factor determining the evaporation rate under certain energy input. The value of h_{lv} is closely related to the material characteristics, which is employed in many reports recently.[5, 6] For example, the application of hydrogel broke the evaporation rate

limitation by introducing three water states in the structure, leading to an optimized h_{lv} which is lower than that of the normal state.[6, 7] Higher evaporation rate is achieved but the energy efficiency remains far below 100% due to the reduction of specific latent heat.

Evaporative Water Enthalpy Evaluation

As indicated in water state investigation within hydrated polymer chains, water can be tuned into three states to reduce the evaporative energy demand. In this work, EEF is proved to be effective in tuning water structure, in turn facilitates the water evaporation through weakening hydrogen bond. Considering the two factors above, the evaporation enthalpy of water during working condition needs to be reevaluated compared to pure water. The dark condition experiment is conducted with glass plate and an area confiner for precise evaluation on mass change. Pure water, pure water with EEF, wetted CAM with EEF are three targets for evaluating the effective evaporation enthalpy. As indicated in Fig. S18, dark condition reference experiment clearly shows a 30% lower (~1511 J/g) evaporation enthalpy with existence of CAM and EEF compared with pure water (2256 J/g). However, no obvious hydration enhancement is shown when compared the evaporative water between EEF enhanced pure water and CAM. Based on EEF-tuned water enthalpy from dark experiment, we estimate the maximum (under voltage of 1800 V) energy conversion efficiency to be 67.1%, 66.8% and 62.9% under 0.5 Sun, 0.75 Sun and 1.0 Sun, respectively. It's worth noting that temperature compromises the EEF effect as shown in the manuscript, indicating dark condition experiment could not precisely reflect the actual evaporation enthalpy under solar illumination.

Table S1. Numerical data of fitted *in-situ* Raman spectra of translation mode.

Translation Mode	Sub-Peak 1					Sub-Peak 2			
	**Aera**	**Center**	**Width**	**Height**		**Aera**	**Center**	**Width**	**Height**
**0V**	1483	92.1	22.3	52.8		6551	159.1	98.3	53.1
**400V**	1212.4	92.4	20.1	48.1		6200.4	163.9	95.4	51.8
**900V**	1126.8	90.3	19.8	45.2		5596.5	157.1	94.4	47.2
**1300V**	2441.3	93.1	23.2	83.6		11746.5	164.5	99.1	94.5
**1800V**	2475.1	90.6	22.5	87.4		11547.9	163.2	98.7	93.2
**2400V**	2468.3	94.0	24.1	81.6		11643.9	165.5	99.9	92.9
**3000V**	2578.6	94.8	26.2	78.4		11511.4	163.6	98.4	93.2
**4000V**	2016.7	92.6	22.8	70.5		10109.0	163.2	99.0	81.4
**4500V**	1729.4	92.0	21.9	62.9		8936.3	161.1	99.4	71.7

Table S2. Numerical data of fitted *in-situ* Raman spectra of OH bend mode.

OH Bend	FWHM	Height	Center
**0V**	193.8	31.7	1656.0
**400V**	246.5	36.1	1659.5
**900V**	329.0	47.2	1666.6
**1300V**	117.8	40.3	1645.3
**1800V**	128.9	35.5	1641.8
**2400V**	139.0	39.8	1652.4
**3000V**	131.9	36.7	1648.9
**4000V**	139.7	35.4	1648.9
**4500V**	162.4	33.8	1655.9

Table S3. Numerical data of fitted *in-situ* Raman spectra of OH Stretch Mode.

OH Stretch	Sub-Peak 1					Sub-Peak 2			
	**Aera**	**Center**	**Width**	**Height**		**Aera**	**Center**	**Width**	**Height**
**0V**	78076.7	3215.7	226.2	275.3		92773.9	3444.0	236.6	312.7
**400V**	76682.5	3223.7	240.3	254.6		76963.0	3452.4	234.2	262.1
**900V**	81717.9	3227.1	246.9	263.9		70670.5	3456.8	225.4	250.1
**1300V**	113540.2	3210.0	216.7	417.9		155864.3	3437.2	242.2	513.3
**1800V**	108652.9	3209.0	214.9	403.2		153496.7	3435.3	243.2	503.4
**2400V**	120719.3	3215.4	225.5	427.0		143560.0	3442.9	236.9	483.3
**3000V**	113910.9	3213.6	218.8	415.3		149560.7	3439.1	240.2	496.6
**4000V**	99017.8	3211.5	218.7	361.1		133981.4	3439.1	242.5	440.7
**4500V**	97159.1	3216.3	228.2	339.6		114981.9	3444.7	238.7	384.3

Table S4. The Lennard-Jones (LJ) parameters used in MD simulations.

Interaction	σ (Å)	ε (kcal/mol)
O-O	3.16435	0.16275
H-H	0.00000	0.00000
O-H	0.00000	0.00000
C-O	3.19000	0.09365
C-H	0.00000	0.00000
C-C	3.43090	0.10500

Supporting Figures

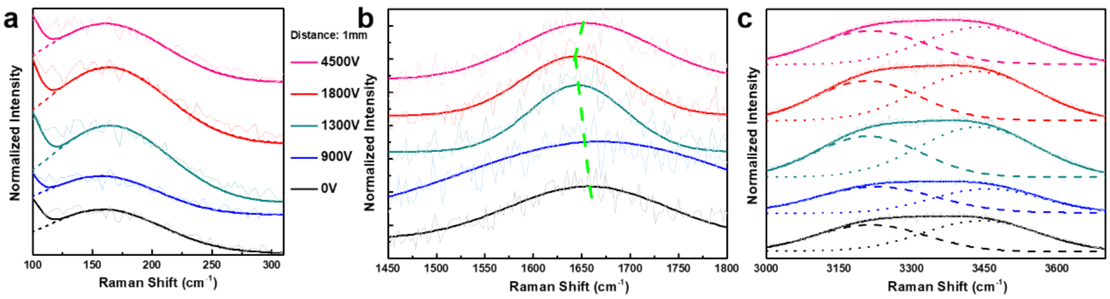

Figure. S1. *In-situ* Raman signals of three modes (a. translation; b. OH bend; c. OH stretch), both original and fitting curves are shown.

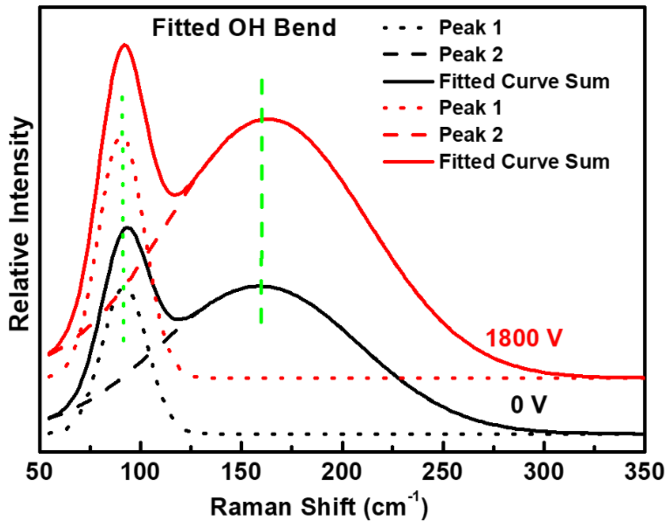

Figure S2. Fitting of translational mode under voltages 0 V and 1800 V. Dashed green line shows non-shifted centers under electrostatic field. Noted that the peak below 100 cm^{-1} is not reliable because this part is in the vicinity of Rayleigh line.

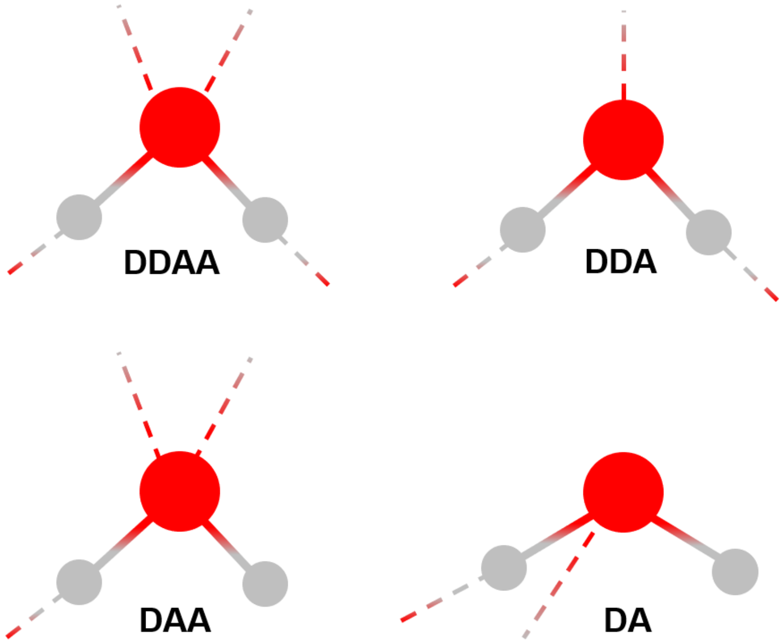

Figure S3. Four kinds of bonding structure containing in OH stretching mode based on classic Gaussian distribution.[9] D means proton donor, where A means proton acceptor. Dotted lines represent weak hydrogen bond among adjacent molecules while solid lines are strong intramolecular bond.

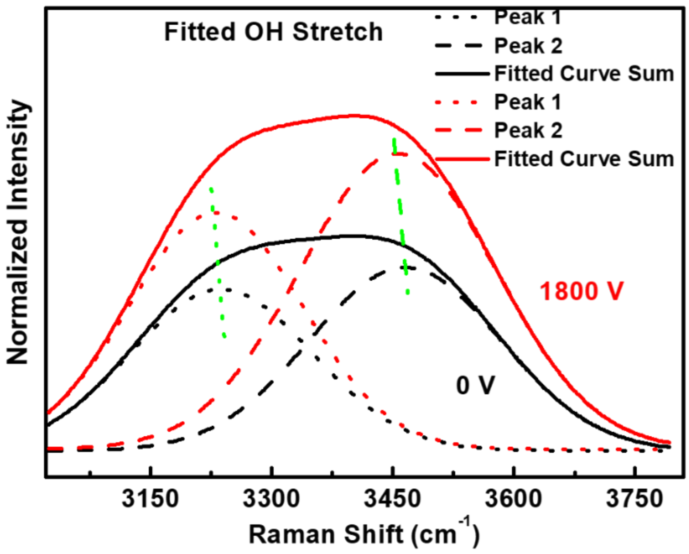

Figure S4. Fitting of OH stretching peak under 0 V and 1800 V. The two fitted sub-peaks contribute to 92% of total OH stretching peak intensity. Dotted green lines show that both sub-peaks exhibit red-shifts to lower frequency under 1800V.

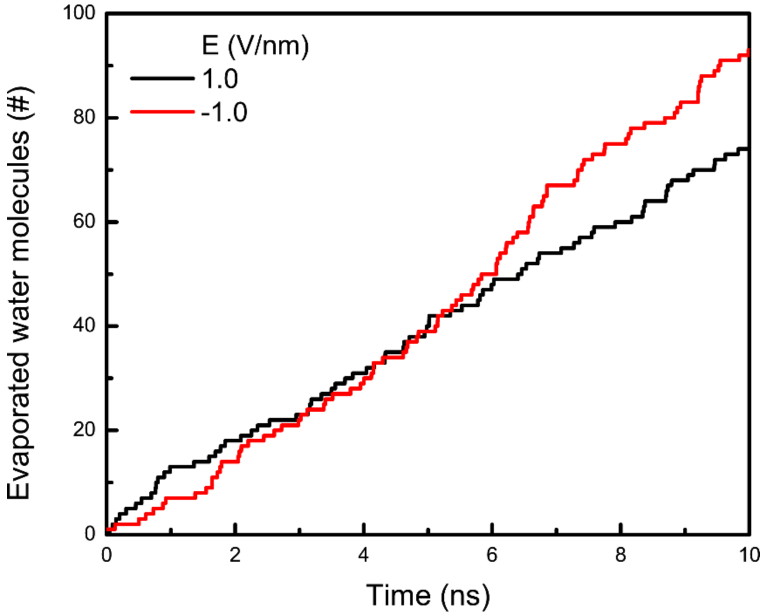

Figure S5. Number of water molecules evaporated from the water layer at temperature T = 316.45 K under EEF with positive direction and negative direction.

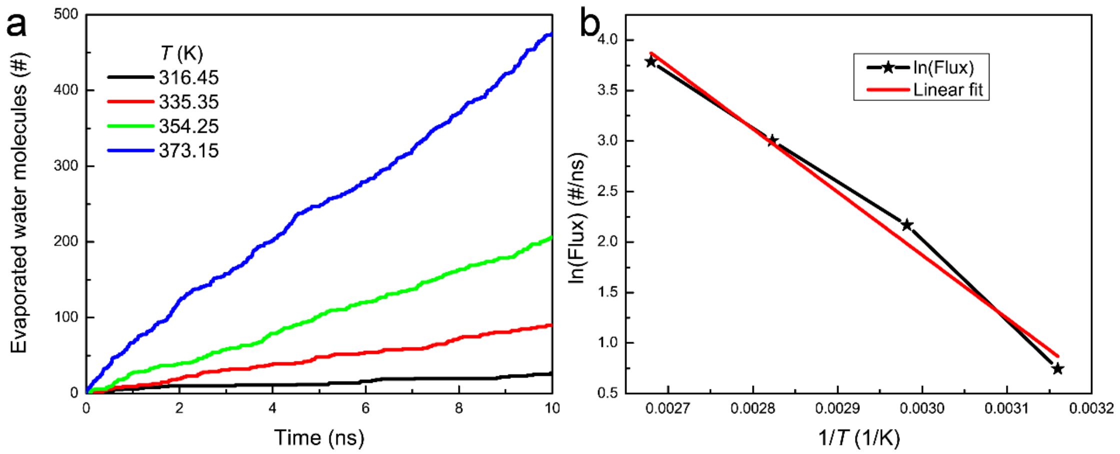

Figure S6. Water evaporation at different temeprature *T*. (a) Number of evaporated water molecules as a function of simulation time at temperatures T = 316.45 K, 335.35 K, 354.25 K, and 373.15 K. (b) The relationship between the evaporated flux and temperature. Data points are extracted from the slopes of the curves in (a). The red curve represents a linear fitting of ln(Flux) to 1/*T*, with the intercept for ln(A) and the slope for $-U_0/k_B$ in Eq. (1). Here, ln(A)=20.63, U_0=12.42 kcal/mol.

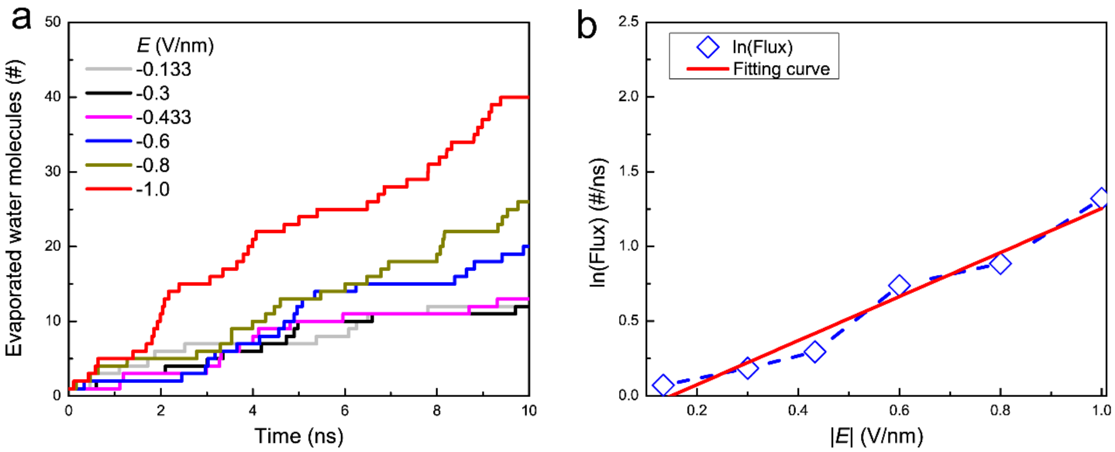

Figure S7. Water evaporation at room temperature T = 298 K under different electrical fields E. (a) Number of evaporated water molecules under different E. (b) The relationship between the evaporated flux and the magnitude of E. Data points are extracted from the slopes of the curves in (a). The red line represents a linear fitting of ln(Flux) to $|E|$, with the slope for α/k_BT in Eq. (1). Here, α = 0.80 kcal/mol/(V/nm).

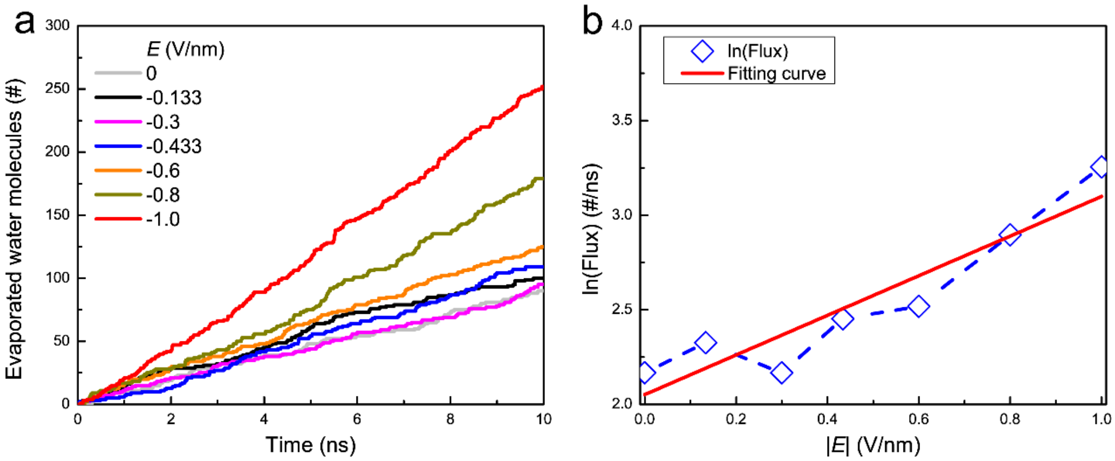

Figure S8. Water evaporation at temperature of T = 335.35 K under different electrical fields E. (a) Number of evaporated water molecules evaporated under different E. (b) The relationship between the evaporated flux and the absolute E. Data points are extracted from slopes of curves in (a). The red line represents a linear fitting of ln(Flux) to $|E|$, with the slope for α/k_BT in Eq. (1). Here, α = 0.70 kcal/mol/(V/nm).

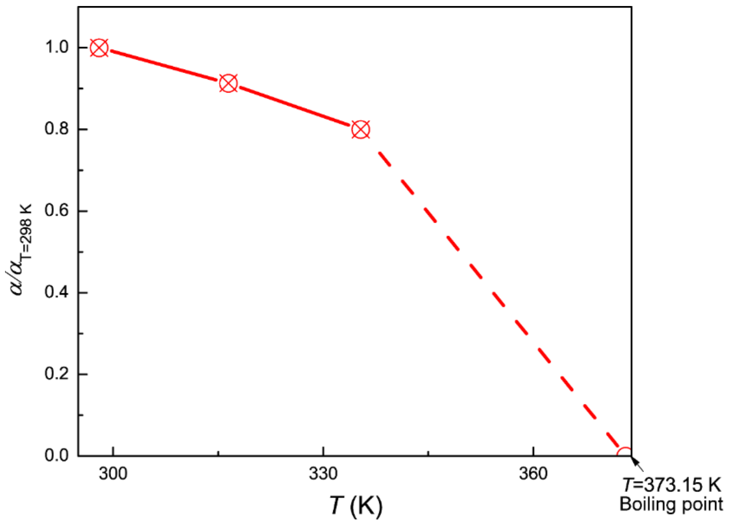

Figure S9. The parameter α as a function of temperature T.

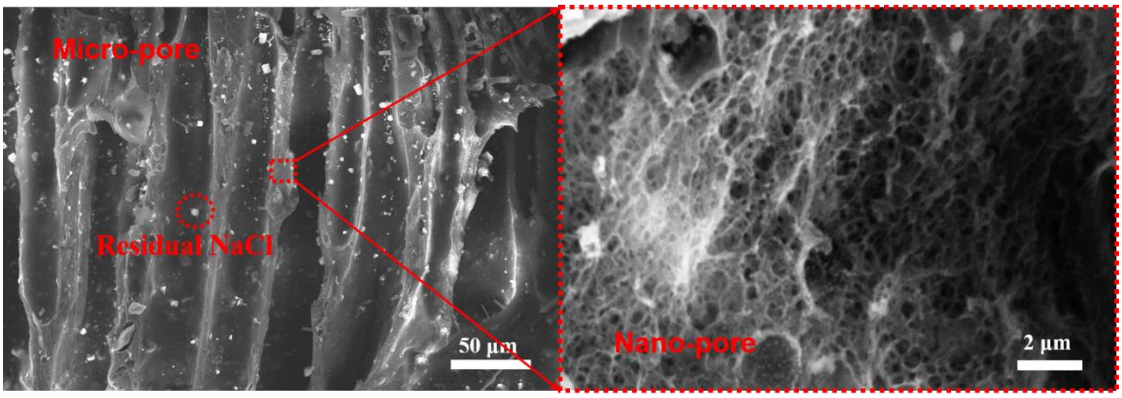

Figure S10. Cross-sectional SEM images of the CAM inner porous structure: a hierarchical porous structure for efficient water transport by capillary interaction. Residual salt crystal coming from the desalination test, showing no structural breakage during operation and wet-dry transition.

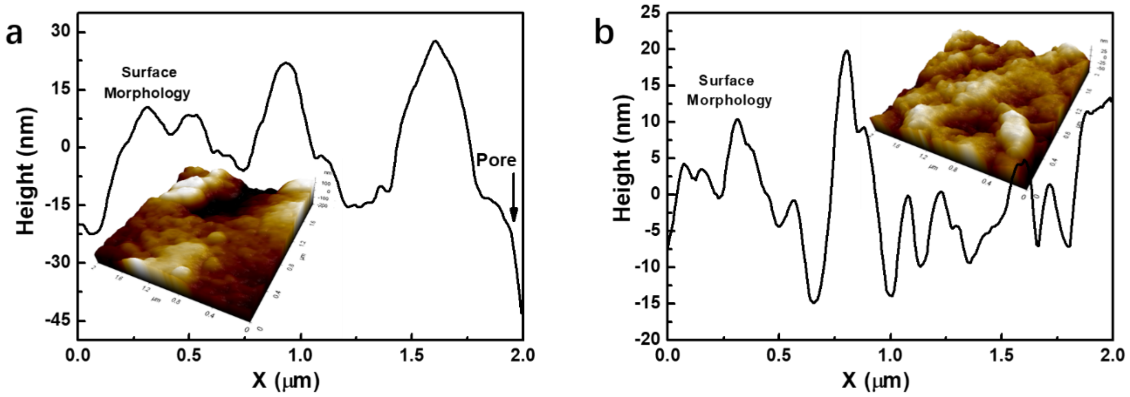

Figure S11. AFM linear scanning of CAM surface topography at (a) dry state, and (b) wet state. Insets: the AFM images. Comparison of AFM images shows a swelling of CAM surface after wetting.

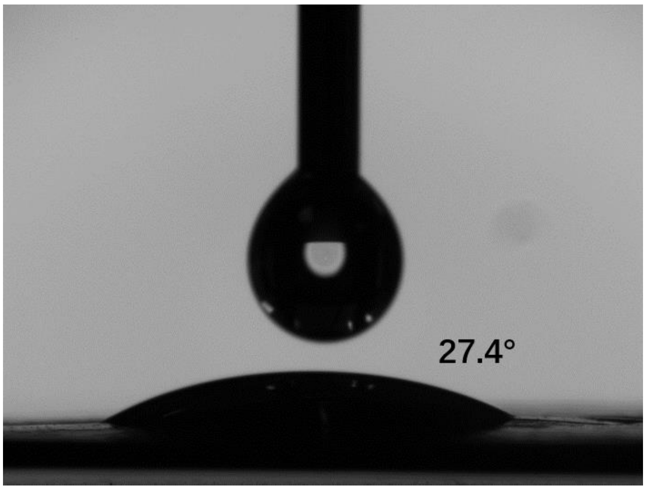

Figure S12. Hydrophilicity test of CAM surface, showing a very hydrophilic surface (contact angle of 27.4°).

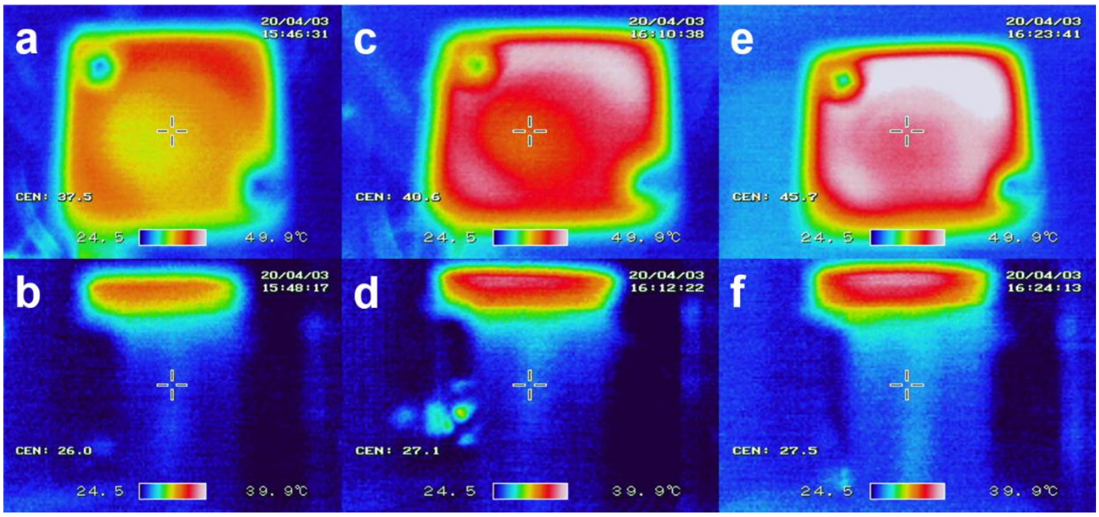

Figure S13. IR images of the water harvesting device under (a-b) 0.5 Sun; (c-d) 0.75 Sun; (e-f) 1.0 Sun. Top views (a,c,e) show high temperature due to heat converted from solar power by photothermal membrane; cross-sectional views (b,d,f) clearly display the outstanding heat localization.

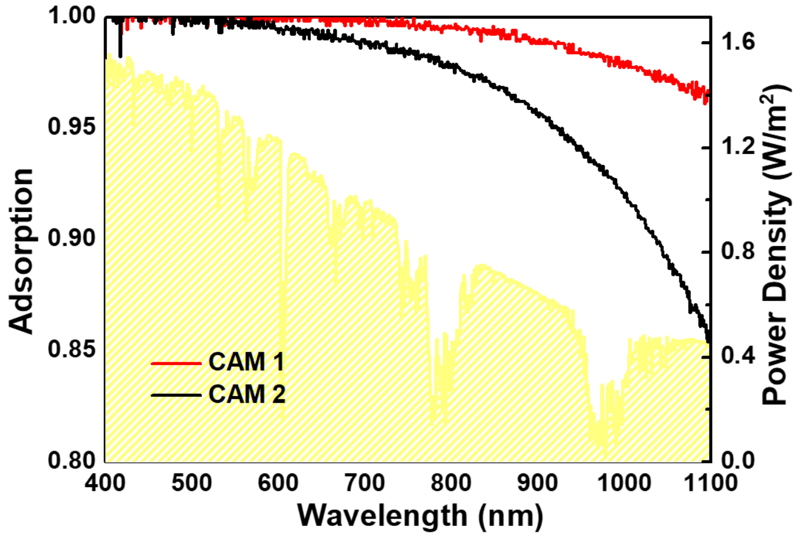

Figure S14. Visible-NIR absorbance of CAM1 and CAM2 with different Li-MXene: cellulose acetate ratios. CAM 1 shows a nearly 100% absorbance in visible range. Background is the standard solar spectrum for reference.

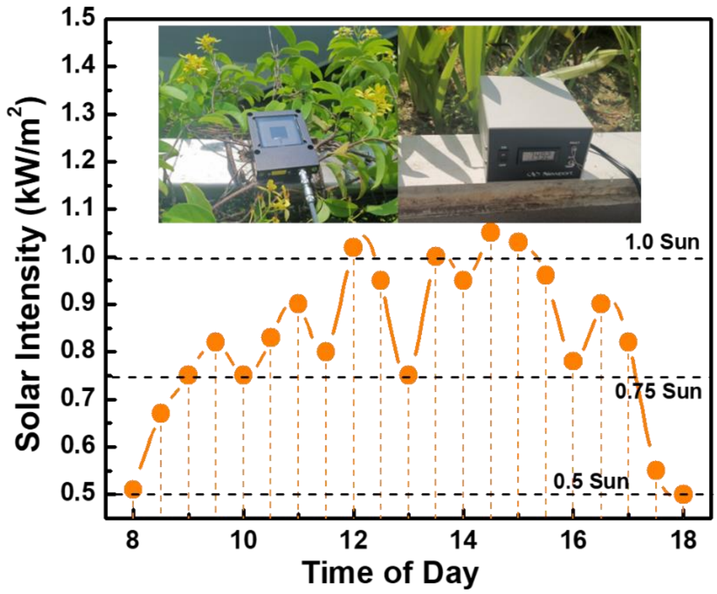

Figure S15. Real-time solar intensity measured in Singapore (N 1°20'54.4"; E 103°40'59.3") from 8 am to 6 pm in March 2020, which indicates a normal natural sunlight range from 0.75 to 0.9 Sun. Inset: images of solar power meter components.

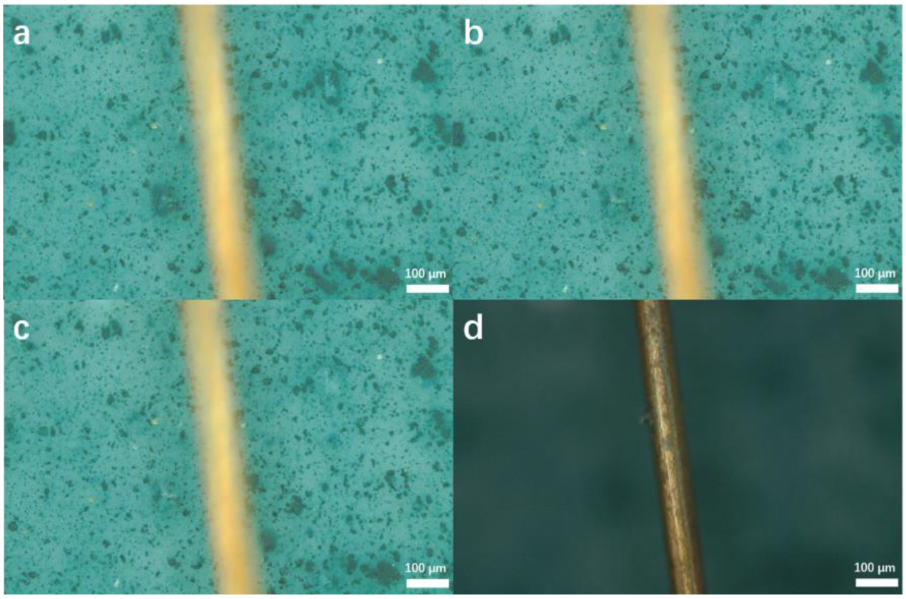

Figure S16. Operando microscopic images of CAM surface under continuously varied external voltages (focusing on water-air interface) of (a) 0V, (b) 1800V, and (c) 2400V. Distance between wire electrode and water surface is 3 mm. There is a negligible change of surface topography under external electrostatic field. (d) Image focusing on a copper wire electrode, showing a diameter of 70 μm.

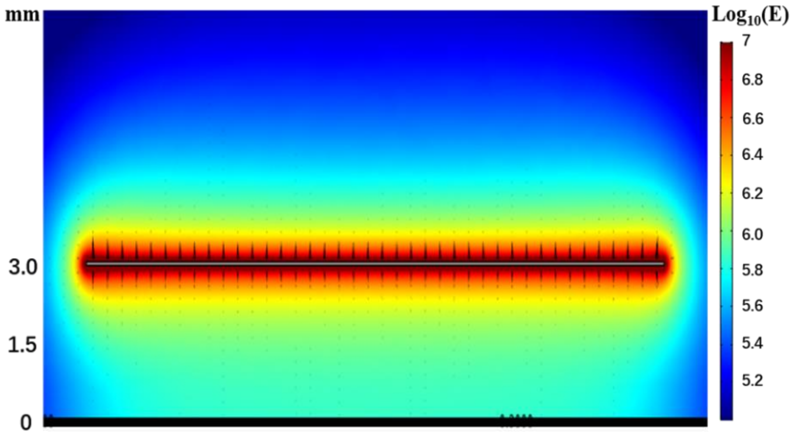

Figure S17. Simulated electrostatic field distribution around copper wire electrode, showing a denser and stronger electrostatic field between electrode and water-air interface.

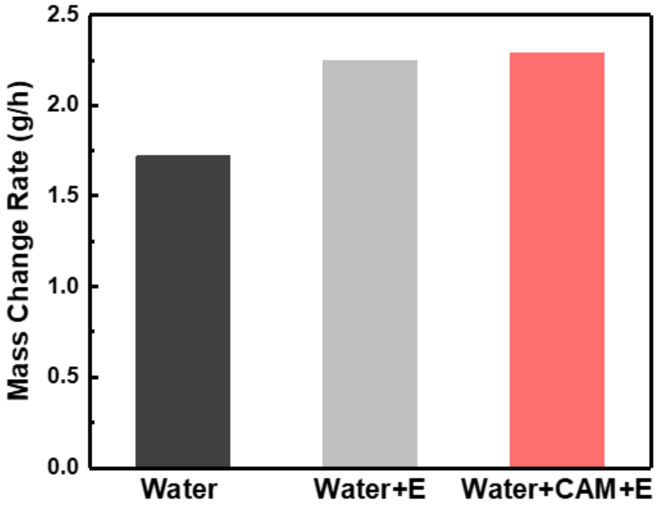

Figure S18. Reference dark experiment for evaluating water enthalpy reduction with existence of CAM and EEF compared to pure water. Pure water evaporative enthalpy is taken as 2256 J/g.

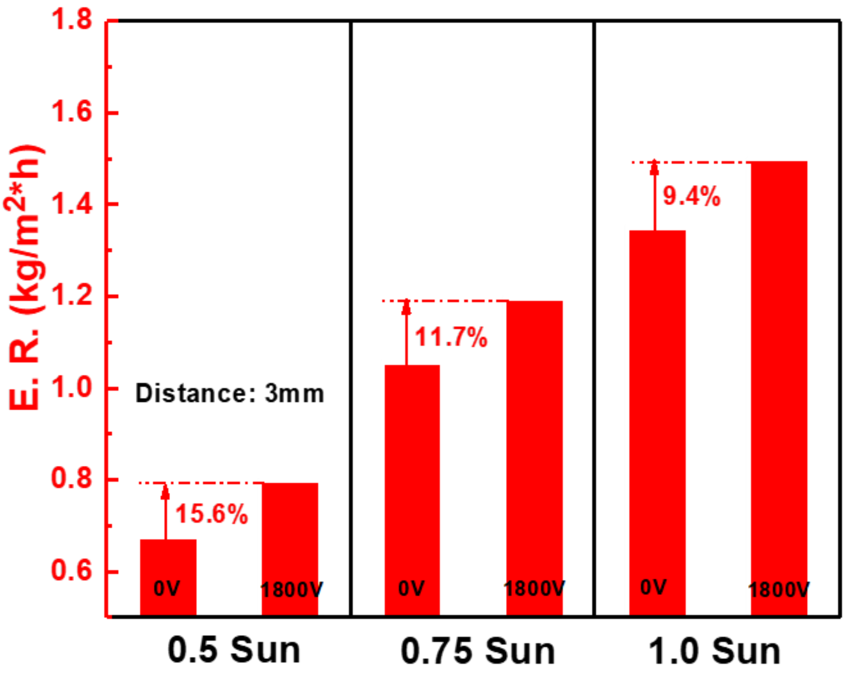

Figure S19. Electrostatic field-enhanced solar stream generation performances under different solar intensities. The enhancement is lower with higher solar intensity.

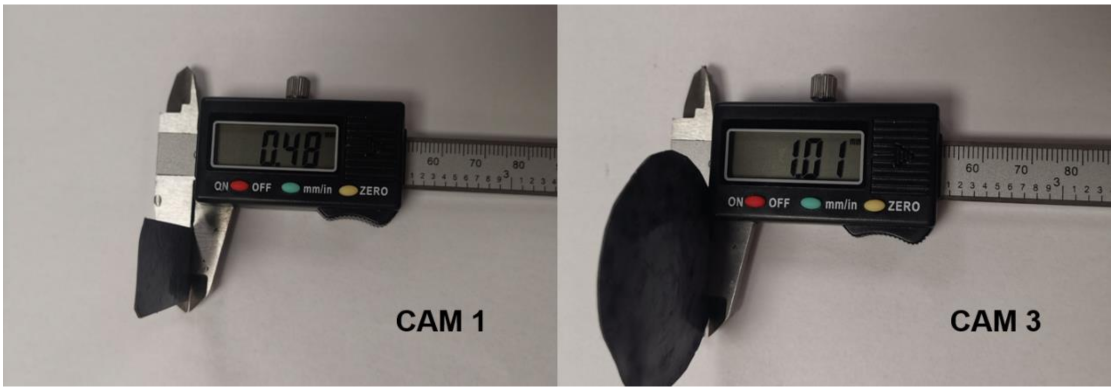

Figure S20. Thickness measurement of CAM 1 (0.48 mm) and 3 (1.01 mm).

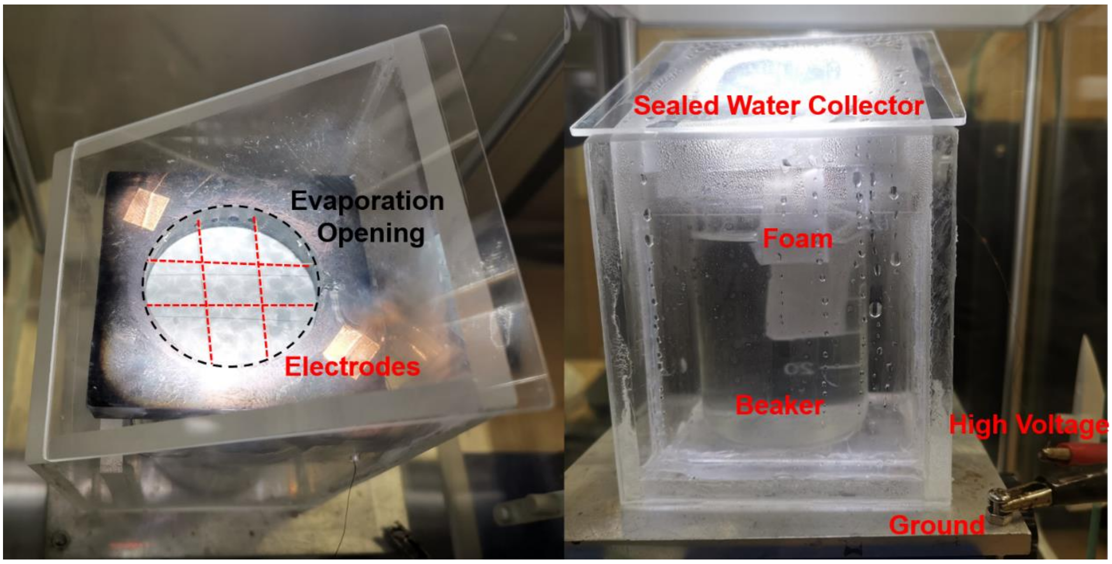

Figure S21. Optical images of designed water harvesting setup from top (left panel) and side views (right panel), components are labeled.

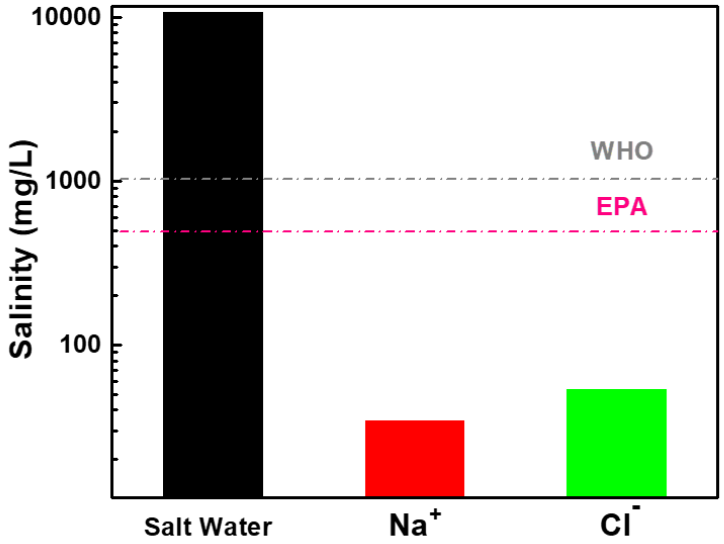

Figure S22. Desalination performance of water harvesting setup in Fig. S20, indicating that drinkable water can be obtained according to two standards, i.e., world health organization (WHO) and US environmental protection agency (EPA).

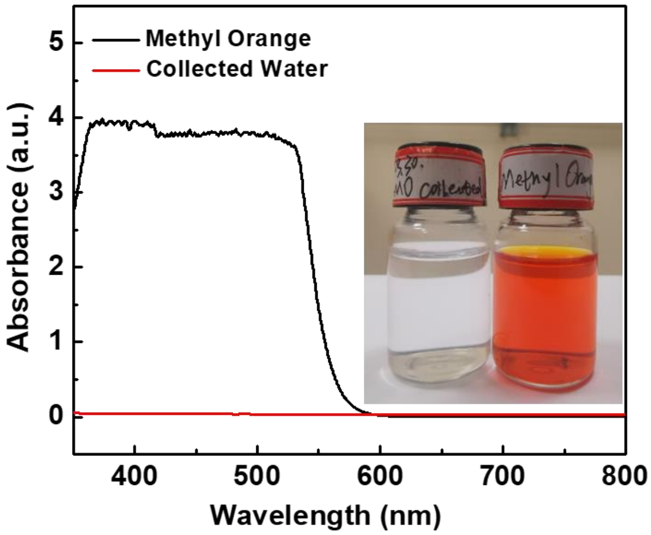

Figure S23. Pollutant (methyl orange) removal during water harvesting, showing a complete purification of collected water from the comparison of visible absorbance spectra.

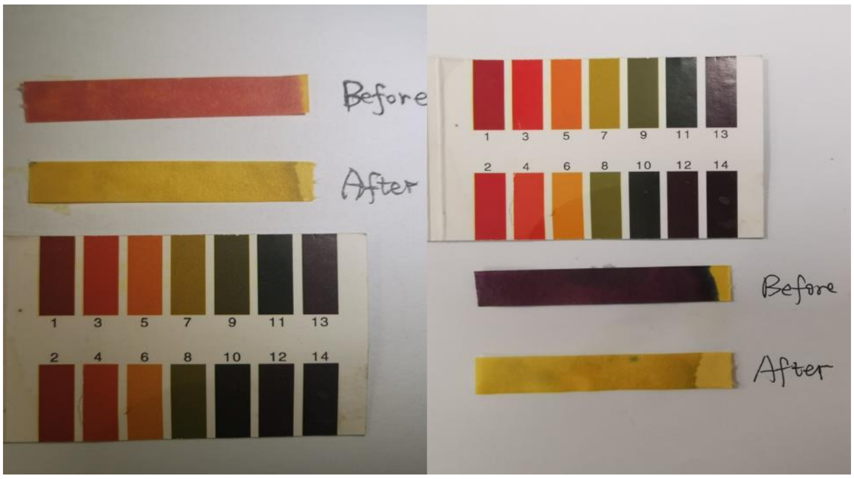

Figure S24. Removal of acidic and alkaline species during water harvesting. Neutral water is collected from the water harvesting. The color code on pH tapes shows the pH value of the tested water.

Supporting Video 1 | Atomistic simulation of water evaporation under E=–0.6 V/nm at temperature T=316.45 K. The video corresponds to the orange line in Fig. 3(b) with the time duration of 10 ns. Atoms colored by red, white, and grey represent oxygen, hydrogen, and carbon, respectively.

Supporting Video 2 | Atomistic simulation of water evaporation under E=–1.0 V/nm at temperature T=316.45 K. The video corresponds to the red line in Fig. 3(b) with the time duration of 10 ns. Atoms colored by red, white, and grey represent oxygen, hydrogen, and carbon, respectively.

Supporting References

[1] X. Li, G. Ni, T. Cooper, N. Xu, J. Li, L. Zhou, X. Hu, B. Zhu, P. Yao, J. Zhu, Joule 2019, 3, 1798.

[2] J. Fei, S. W. Koh, W. Tu, J. Ge, H. Rezaeyan, S. Hou, H. Duan, Y. C. Lam, H. Li, Adv. Sustain. Syst. 2020, 4, 2000102.

[3] L. Zhou, Y. Tan, J. Wang, W. Xu, Y. Yuan, W. Cai, S. Zhu, J. Zhu, Nat. Photonics 2016, 10, 393.

[4] R. Li, L. Zhang, L. Shi, P. Wang, ACS Nano 2017, 11, 3752.

[5] Y. Guo, F. Zhao, X. Zhou, Z. Chen, G. Yu, Nano Lett 2019, 19, 2530.

[6] Y. Guo, X. Zhao, F. Zhao, Z. Jiao, X. Zhou, G. Yu, Energy Environ. Sci. 2020, 13, 2087.

[7] a) X. Zhou, Y. Guo, F. Zhao, G. Yu, Acc Chem Res 2019, 52, 3244; b) X. Zhou, F. Zhao, Y. Guo, B. Rosenberger, G. Yu, Sci. Adv. 2019, 5, eaaw5484; c) X. Zhou, F. Zhao, Y. Guo, Y. Zhang, G. Yu, Energy Environ. Sci. 2018, 11, 1985.

[8] Y. Guo, X. Zhou, F. Zhao, J. Bae, B. Rosenberger, G. Yu, ACS nano 2019, 13, 7913.

[9] Q. Sun, Vibrational Spectroscopy 2009, 51, 213.
